# Supplementary material for: MetaRibo-Seq measures translation in microbiomes
Source: Nat Commun. 2020 Jun 29;11:3268. doi: 10.1038/s41467-020-17081-z (PMC7324362; doi:10.1038/s41467-020-17081-z)
Supplement: Supplementary file 10 — Supplementary Data 7 [file 41467_2020_17081_MOESM10_ESM.zip › File2/Confidence_High_Taxonomy/322823_out.krona.html]

Javascript must be enabled to view this page.

members
magnitude
magnitudeUnassigned
count
unassigned
taxon
rank

322823\_out

2

2
2
superkingdom

2
1224
phylum

2
subphylum
68525

2
class
29547

order
213849
2

72294
family
2

genus
194
2

2
species
199

SRS016086\_contig\_number\_23907SRS143022\_contig\_number\_11828
